# Effects of vacuum and high-oxygen modified atmosphere packaging on physico-chemical and microbiological properties of minced water buffalo meat

**DOI:** 10.5713/ajas.18.0391

**Published:** 2018-08-27

**Authors:** Rahimeh Jaberi, Güzin Kaban, Mükerrem Kaya

**Affiliations:** 1 Department of Food Engineering, Faculty of Agriculture, Atatürk University, Erzurum 25240, Turkey

**Keywords:** Minced Water Buffalo Meat, Vacuum, HiOx-MAP, Color, *Pseudomonas*

## Abstract

**Objective:**

In this study, the effects of vacuum (VP) and high-oxygen modified atmosphere (80% O_2_+20% CO_2_) packaging (HiOx-MAP) on physico-chemical and microbiological properties of minced water buffalo meat were investigated.

**Methods:**

After minced meat preparation, samples were packaged under VP and HiOx-MAP and stored at 2°C±0.5°C for 14 days. Samples taken on certain days were subjected to total aerobic mesophilic bacteria, total aerobic psychrotrophic bacteria, lactic acid bacteria, *Pseudomonas*, Enterobacteriaceae and yeast-mold counts as well as pH, color (L*, a*, and b*) and thiobarbituric acid reactive substances (TBARS) analyses.

**Results:**

In minced water buffalo meat packaged under HiOx-MAP, TBARS value exceeded 1 mg malondialdehyde/kg on the 4th day of the storage. In VP samples, TBARS value remained close to initial TBARS value during storage. According to the findings, a* value was determined to be high in the HiOx-MAP samples within initial days of the storage. However, no significant changes in a* value were observed in VP samples during storage. In contrast, the mean value of L* was detected as higher in HiOx-MAP sample than VP samples. The count of psychrotrophic bacteria increased more than that of mesophilic bacteria during storage. The growth of Enterobacteriaceae and *Pseudomonas* was delayed in both the packaging methods. However, lactic acid bacteria exhibited more growth in VP samples compared to MAP samples.

**Conclusion:**

The lipid oxidation proceeded faster than expected in minced water buffalo meat packed with HiOx-MAP method. This situation adversely affected the a* value. On the other hand, similar microbiological results were obtained in both packing methods.

## INTRODUCTION

Water buffalo (*Bubalus bubalis*) meat is different from beef meat since it contains low intermuscular fat, cholesterol, calories and high essential amino acids, biological value and iron [1–3]. Also, it is considered as a good potential raw material due to its high protein and low-fat content [2]. Buffalo meat is used as raw material in fresh meat products such as patties [3], nuggets [4], burgers [5], in fermented sausages [6,7] and in emulsified meat products [8,9]. Buffalo meat is also used in the production of raw cured or salted meat products made from whole muscle such as pastırma [10] and Bresaola [11].

Minced meat provides a good medium for growth of different microorganisms due to increasing the surface area through the mincing process [12]. At the same time, minced meat is more prone to pigment and lipid oxidation than intact muscle cuts [13]. Because of these, lipid oxidation and microbial count are also considered as important quality criteria in minced meat. The spoilage of minced meat depends on meat composition, the hygienic practices during process and the storage time and temperature [12]. Also, the packaging method is an important factor in the rate of deteriorative changes [14].

Minced meat has a short shelf life under aerobic conditions. The spoilage microbiota of minced meat under these conditions is dominated by *Pseudomonas* and to a lesser extent by Enterobacteriaceae [15]. High levels of oxygen promote lipid oxidation, which causes rancidity in meat, and discolorization [13]. In contrast, since oxygen is eliminated in vacuum packaging, a more stable color and longer shelf life is provided in vacuum packaging as a result of inhibition of the growth of aerobic bacteria and limiting oxidation. Moreover, the vacuum packaging does not contribute to the color demanded by the consumer [16,17]. The conversion of oxymyoglobin to metmyoglobin causes meat’s color to turn from red to brown [17, 18]. In vacuum-packaged minced meat and similar products, metmyoglobin is the dominant pigment. Because of their dark drab brownish color, vacuum-packaged minced meat and similar products are not preferred by the consumers. Moreover, the use of vacuum package may cause undesirable sensory changes such as acid taste and cheesy odor because of the growth of the anaerobic spoilage microorganism [17]. On the other hand, modified atmosphere packaging (MAP) to ensure the microbiological shelf-life and desirable color of the meat is the most popular treatment of packaging [13,14,16,17]. In fresh meat products, the gas combination with 80% O_2_+20% CO_2_ is widely used in the treatment of high-oxygen modified atmosphere packaging (HiOx-MAP) [14].

There are a few studies about cold storage of minced buffalo meat in which the effects of various additives were investigated on the shelf life of the product stored under aerobic conditions in cold [19–21]. No studies have been found on the shelf life and qualitative properties of minced water buffalo meat in vacuum and under modified atmosphere conditions. The aim of this study, therefore, is to determine the effects of vacuum and high-oxygen modified packaging methods on physico-chemical and microbiological properties of minced water buffalo meat. For this aim, minced water buffalo meat was packaged under vacuum and high-oxygen modified atmosphere (80% O_2_+20% CO_2_) conditions and stored at 2°C±0.5°C for 14 days. Samples taken at the certain days of storage were subjected to total aerobic mesophilic bacteria, total aerobic psychrotrophic bacteria, lactic acid bacteria, *Pseudomonas*, Enterobacteriaceae and yeast-mold counts as well as pH, color (L*, a*, and b*) and thiobarbituric acid reactive substances (TBARS) analyses.

## MATERIALS AND METHODS

### Material

For the preparation of the minced water buffalo meat, buffalo meat (*Bubalus bubalis*) was taken from the shoulder of the adult female (about 3 to 4 years of age) buffalo carcass conditioned at 4°C±1°C for 24 h obtained from the local slaughterhouse (Meat and Milk Institution, Erzurum, Turkey). In the study, meat obtained from one carcass was divided into three groups and the experiment was conducted as three replications.

### Preparing and packaging of minced meat

Water buffalo meat was minced using a meat grinder (Laska, Austria). The minced water buffalo meat (15% fat) was prepared in 160 g portions and packed (Multivac, Germany) under vacuum or high-oxygen modified atmosphere (80% O_2_+20% CO_2_) conditions; for packaging polyamide/polyethylene (15×25 cm, PA/PE) (3-seal bags GB 70) material (O_2_ permeability 40 cm^3^/m^2^/d·atm·23°C; N_2_ permeability 24 cm^3^/m^2^/d·atm·23°C; CO_2_ permeability 1,454 cm^3^/m^2^/d·atm·23°C; and water vapor permeability <3 g/m^2^/d·atm·23°C), (Südpack Verpackungen GmbH Co, Ochsenhausen, Germany) were used. The ratio of gas to minced meat was approximately 2:1. The food grade gas mixture was supplied by Karbogaz (İstanbul, Turkey). The samples packaged in vacuum and under high-oxygen modified atmosphere (80% O_2_+20% CO_2_) conditions were stored at 2°C±0.5°C for 14 days. The analyses were performed on certain days of storage.

### Microbiological analysis

Plate Count Agar (PCA, Oxoid Limited, Hampshire, UK) was used for the enumeration of total aerobic mesophilic bacteria and the plates were incubated aerobically at 37°C for 48 h. For total psychrotrophic bacteria, PCA (Oxoid, UK) was used as well and the count was enumerated at 7°C for 10 days. *Pseudomonas* were determined on CFC Agar (*Pseudomonas* Agar Base CM 0559-Oxoid) with Selective Agar Supplement (SR0103, Oxoid, UK) and the plates were incubated under aerobic conditions at 25°C for 48 h. Violet Red Bile Dextrose Agar (VRBD Agar, Merck KGaA, Darmstadt, Germany) was used for Enterobacteriaceae and the plates were incubated 48 h at 30°C under anaerobic conditions (Anaerocult A, Merck, Germany). Also, for lactic acid bacteria, Man Rogosa Sharpe Agar was used (MRS, Oxoid, UK) at 30°C for 48 h under anaerobic conditions. Rose-Bengal Chloramphenicol Agar (RBC, Merck, Germany) was used to determine the count of yeast/mold and plates were incubated at 25°C for 5 days under aerobic conditions.

### pH determination

Ten grams of minced meat was added to 100 mL of distillated distilled water. After homogenization for 1 min with Ultra-Turrax (IKA Werk Tp 18; IKA-Werke GmbH & Co. KG, Staufen, Germany), pH value was determined using a pH meter (ATI ORION 420A; ATI Orion Company, Boston, MA, USA).

### Determination of thiobarbituric acid reactive substances (TBARS)

TBARS value was determined by Lemon’s [22] method. The result was given in mg malondialdehyde (MDA)/kg.

### Determination of color values

The color values (L*, a*, and b*) were measured immediately after opening the package. In determining the color values, a Minolta colorimeter (CR-200, Minolta Co., Osaka, Japan) was used for evaluation ([L* = 0, black; L* = 100, white (darkness/lightness); a*; +a* = red, −a* = green and b*; +b* = yellow, −b* = blue).

### Statistical analysis

Packaging method (vacuum and high-oxygen modified atmosphere) and storage period (0, 2, 4, 6, 8, 10, 12, and 14 days) were considered as factors. The experiment was replicated three times and carried out in 2×8 factorial design according to a completely randomized design. The obtained data were subjected to variance analysis and Duncan’s multiple range test was used for comparing the means to find out the effect of packaging method and storage period on various parameters [23].

## RESULTS AND DISCUSSION

### pH value

The storage time had a very significant effect (p<0.01) on the pH value of minced buffalo meat, however, the interaction of packaging method and packaging method×storage time had no significant effect (p>0.05) on pH value of the samples. During storage, the lowest pH value was measured on the 0th day as 5.65 and the highest pH value was determined on the 8th day as 5.75 (Table 1). According to this result, limited proteolytic activity and good growth of lactic acid bacteria prevented the increase of pH during storage in both VP and HiOx-MAP samples. Moreover, inhibiting the growth of *Pseudomonas* and Enterobacteriaceae during storage has contributed to this result.

### Thiobarbituric acid reactive substances value

**Figure 1:**
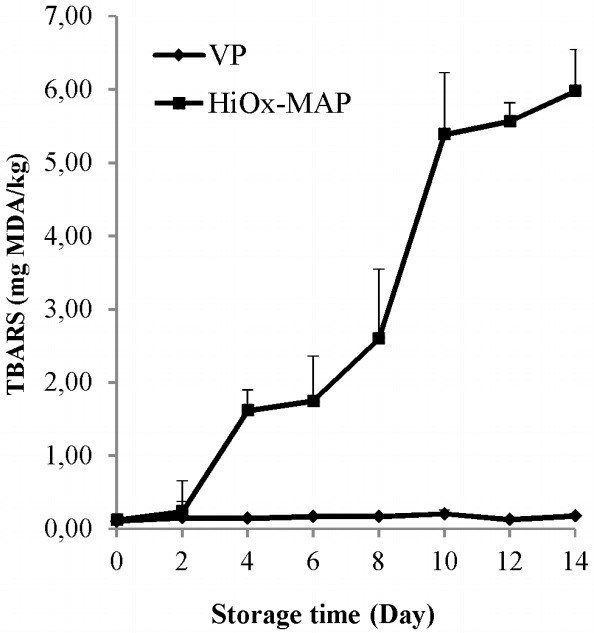
Effects of vacuum packaging (VP) and high-oxygen modified atmosphere packaging (HiOx-MAP, 80% O_2_+2% CO_2_) on thiobarbituric acid reactive substances (TBARS) value of minced water buffalo meat at 2°C±0.5°C for 14 days.

TBARS value in HiOx-MAP samples had a very high average compared to the VP samples (Table 1). The interaction of packaging method×storage time had a very significant effect (p<0.01) on TBARS value. In other words, the most important change and significant increases occurred in HiOx-MAP samples during storage (Figure 1). However, the level of lipid oxidation for VP and HiOx-MAP samples remained the same up to 2 days. The limit for TBARS in fresh meat products is 1 mg MDA/kg, however, in HiOx-MAP samples, TBARS value was measured as 1.62 mg MDA/kg on the 4th day and observed to be over 5.0 mg MDA/kg on the 10th day (Figure 1). According to Lauzurica et al [24], modified atmosphere packaging with high levels of oxygen encourages the lipid oxidation of fresh meat and meat products. These results are also in agreement with previous studies [24–26] that used high levels of oxygen in modified atmosphere packaging which increased TBARS value because of lipid oxidation. Similarly, Lorenzo and Gomez [27] reported that vacuum application decreased lipid oxidation in foal meat samples but the samples packaged with high oxygen levels had the highest TBARS value. Murphy et al [28] reported that after application of vacuum and MAP (80% O_2_+20% CO_2_) on beef steaks for 14 days, TBARS values in vacuum-packaged samples were below 0.2 mg MDA/kg and approximately 1 mg MDA/kg in MAP samples.

### Color values

**Figure 2:**
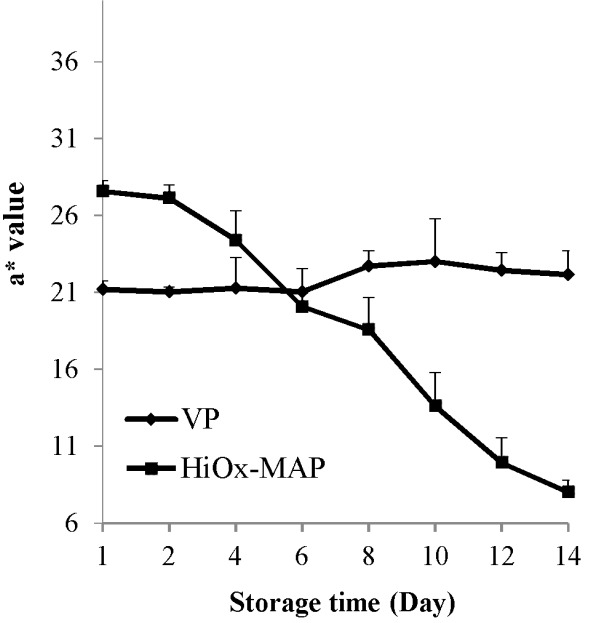
Effects of vacuum packaging (VP) and high-oxygen modified atmosphere packaging (HiOx-MAP, 80% O_2_+20% CO_2_) on a* value of minced water buffalo meat at 2°C±0.5°C for 14 days.

**Figure 3:**
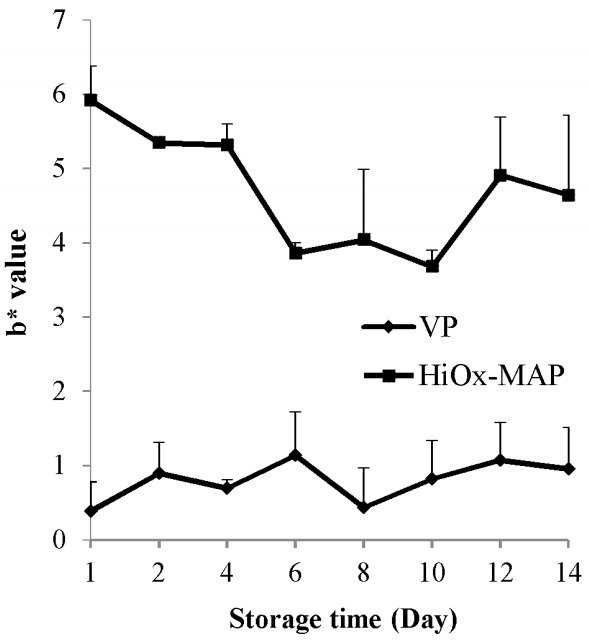
Effects of vacuum packaging (VP) and high-oxygen modified atmosphere packaging (HiOx-MAP, 80% O_2_+20% CO_2_) on b* value of minced water buffalo meat at 2°C±0.5°C for 14 days.

As shown in Table 2, the packaging method had a very significant effect (p<0.01) on L*, a*, and b* values. Storage time had a very significant effect (p<0.01) on a* value, while a significant effect (p<0.05) was observed on L* and b* values of minced water buffalo meat. The existence of high oxygen on MAP packaging samples during storage resulted in a high L* value when compared to VP samples. The highest mean a* value was in VP method. It was observed that the initial average of a* value was 24.40 and steadily decreased to 15.11 up to 14 day of storage due to oxidation of color pigments during storage (Table 2). Several researchers have reported that myoglobin changes to bright cherry-red oxymyoglobin due to high levels of oxygen in modified atmosphere packaging [29,30], and measuring of a* value is a significant parameter for oxidation on meat [25]. Likewise, our results showed also that an increase in lipid oxidation was accompanied by discoloration. Packaging with high oxygen in MAP had resulted in a bright cherry red color in the first few days and oxygen progressively reduced to metmyoglobin during the storage period [31]. Because of oxygen decrease and metmyoglobin formation, a decrease in L* value in vacuum-packaged samples was observed (Table 2). The interaction of packaging method and storage time had a very significant effect (p<0.01) on a* and b* values. It had no significant effect (p>0.05) on L* value of minced water buffalo meat. The a* value showed a slight increase after the 6th day in vacuum packaging and no significant changes were observed in the other days of storage (Figure 2). According to this result, there is more color stability in vacuum-packed meat as reported by other studies [27]. The mean b* value in HiOx-MAP packaging method was higher compared to VP samples. During storage time, b* values changed between 2.24 and 3.16 (Table 2). Likewise a* value, b* value showed stability in vacuum packaging as well. As it can be seen in Figure 3, b* value showed less stability in samples subjected to HiOx-MAP when compared to vacuum packaging (Figure 3). Esmer et al [32] reported that a* value of minced beef meat decreased significantly in MAP (30% CO_2_+70% O_2_). Also, Berruga et al [33] reported that vacuum packaging of lamb meat samples had resulted in lower L* value compared to MAP samples and higher a* and b* values were for MAP (80% O_2_+ 20% CO_2_), but a* value in this gas composition quickly decreased in comparison with vacuum-packaged samples.

### Microbiological analysis

**Figure 4:**
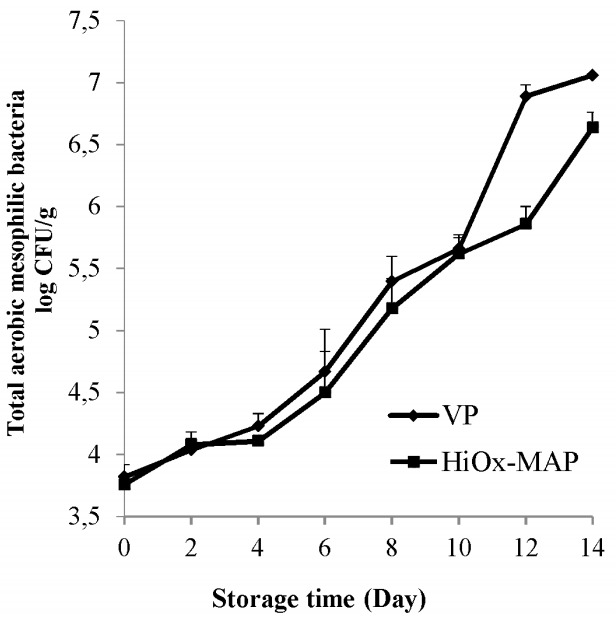
Effects of vacuum packaging (VP) and high-oxygen modified atmosphere packaging (HiOx-MAP, 80% O_2_+20% CO_2_) on total aerobic mesophilic bacteria count of minced water buffalo meat at 2°C±0.5°C for 14 days. CFU, colony-forming unit.

The packaging method, storage time and the interaction of packaging method and storage time had a very significant effect (p<0.01) on total aerobic mesophilic bacteria count of minced water buffalo meat (Table 3). As can be seen from Table 3, the initial count (at the 0 day) for total aerobic mesophilic bacteria was 3.79 log colony-forming unit (CFU)/g, representative of good quality. During chilled storage, the count of total aerobic mesophilic bacteria increased more rapidly after the 10th day in VP samples (Figure 4). According to International Commission on Microbiological Specifications for Foods [34], the upper microbial limit of acceptability for meat is 7 log CFU/g. In the study, total aerobic mesophilic bacteria reached 7 log CFU/g on 14th days, in contrast, this count was not reached in HiOx-MAP samples at the same days (Figure 4). As can be seen Table 4, HiOx-MAP samples had a lower mean count of total aerobic mesophilic bacteria than VP samples. The finding that the existence of CO_2_ in MAP samples had an antimicrobial effect on the growth of the microorganism is in agreement with [35] who reported slow growth of total aerobic mesophilic bacteria in MAP (70% O_2_+30% CO_2_) application on minced beef compared to vacuum-packaged samples.

Packaging method had a significant effect (p<0.05) and storage time had a very significant effect (p<0.01) on psychrotrophic bacteria in minced water buffalo meat. However, the interaction of the two factors had no significant effect (p> 0.05) on psychrotrophic bacteria. HiOx-MAP samples had a highest average of psychrotrophic bacteria count than vacuum samples (Table 3). These results showed that vacuum packaging inhibited the growth of Gram-negative psychrotrophic bacteria (especially *Pseudomonas*) compared to HiOx-MAP samples. In the same fashion, Değirmencioğlu et al [35] also reported that psychotropic aerobic bacteria are inhibited more in minced meat under vacuum packaging when compared to MAP applications. However, it was also reported in another study conducted on foal meat that vacuum and HiOx-MAP applications gave similar results [27]. The average count of psychrotrophic bacteria increased during storage time and this increase was above 1×10^6^ CFU/g on the 10th day (Table 3). The growth of psychrotrophic bacteria in meat at cold temperature depends on such factors as the initial number of psychrotrophic microorganisms, pH value, storage temperature, and packaging method [36].

The results of *Pseudomonas* counts are shown in Table 3. Packaging method and storage time had a significant effect (p<0.05). However, the interaction of packaging method and storage time had no significant effect (p>0.05). The growth of *Pseudomonas* was inhibited in both packaging methods. However, HiOx-MAP application gave higher average value than the vacuum application. But, the difference between two averages was not more than 0.5 log unit (Table 3). In a similar way, Lorenzo and Gomez [27] also reported that *Pseudomonas* counts in foal meat under HiOx-MAP application (4.70 log CFU/g) was higher than that of vacuum packaging (4.41 log CFU/g) and the difference between the two applications was lower than 0.5 log CFU/g, as in our study. In contrast, Değirmencioğlu et al [35] reported that there were no statistically significant differences between vacuum and 70% O_2_+30% CO_2_ applications in terms of *Pseudomonas* counts in minced meat samples stored at 4°C for 7 days. Despite the variety of initial microflora in the meat, *Pseudomonas* spp. survive predominantly under aerobic conditions at refrigerator storage. The mean count of *Pseudomonas* was enumerated as 3.51 log CFU/g (0 day) at the beginning of storage while it was 3.28 log CFU/g at the 10th day. The difference between these two values was found to be statistically insignificant. The mean count of *Pseudomonas* showed a slight change during storage and remained at 10^3^ CFU/g. It has been reported that the use of 20% or more carbon dioxide in MAP packaging plays an important role in inhibiting the growth of these microorganisms [29]. Similarly, there were no significant increases reported in *Pseudomonas* counts of vacuum-packaged samples in another study [35].

**Figure 5:**
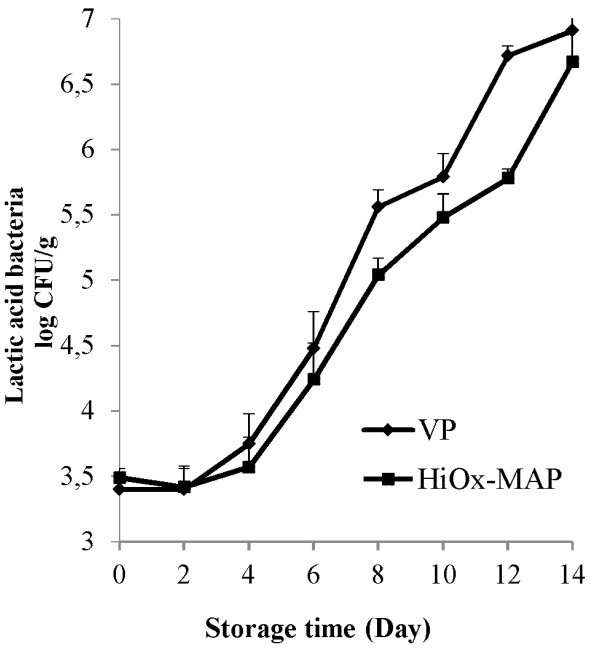
Effects of vacuum packaging (VP) and high-oxygen modified atmosphere packaging (HiOx-MAP, 80% O_2_+20% CO_2_) on lactic acid bacteria count of minced water buffalo meat at 2°C±0.5°C for 14 days. CFU, colony-forming unit.

The average initial count of lactic acid bacteria was 3.44 log CFU/g. There was no significant statistical difference up to the 4th day of storage compared to 6.80 log CFU/g on the 14th day of storage. On the other hand, the mean count of lactic acid bacteria was found higher in VP samples compared with HiOx-MAP samples (Table 3). Berruga et al [33] found that lactic acid bacteria showed a better growth in vacuum packaged lamb meat samples compared to MAP (80% O_2_+20% CO_2_) samples during storage. Also, Değirmencioğlu et al [35] reported similar results that agree with these results. As shown in Figure 5, VP samples had the highest lactic acid bacteria count compared to HiOx-MAP samples from the 4th day of storage and the greatest difference of lactic acid bacteria between packaging methods was found with 1 log unit on the 12th day. At the end of storage, lactic acid bacteria count under vacuum packaging were relatively higher (Figure 5). According to these results, these facultative anaerobic bacteria can grow better in anaerobic environments despite their ability to grow under high carbon dioxide concentrations. According to Berruga et al [33], vacuum application of lamb meat resulted in a faster growth of lactic acid bacteria than MAP (80% O_2_+20% CO_2_) method. Lee and Yoon [37] also reported an increase of lactic acid bacteria count in vacuum application of ground beef compared to MAP samples.

Packaging method and storage time had a very significant effect (p<0.01) on Enterobacteriaceae in minced water buffalo meat. In contrast, the interaction of packaging method and storage time had no significant effect (p>0.05). The highest average count was observed in VP samples (Table 3). It was also reported in other studies that MAP applications are more effective on Enterobacteriaceae counts [27,35,38]. Members of this family, which are seen as an indicator of hygienic conditions, show facultative anaerobic character and are inhibited significantly even at 20% carbondioxide concentrations [39].

**Figure 6:**
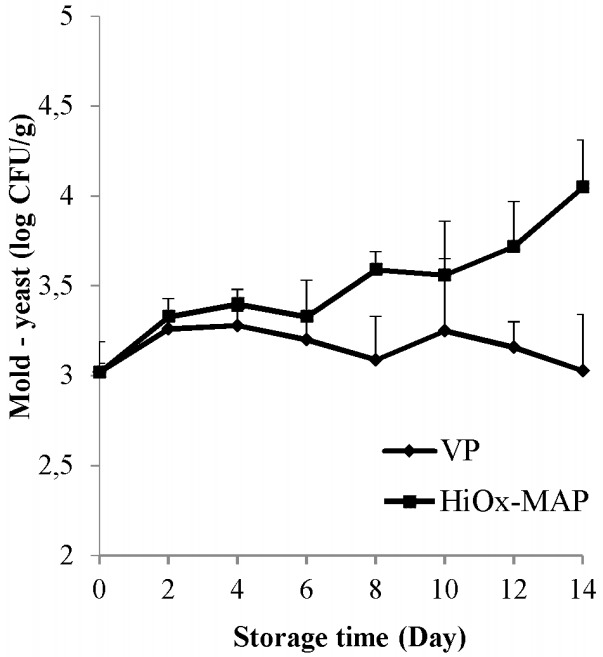
Effects of vacuum packaging (VP) and high-oxygen modified atmosphere packaging (HiOx-MAP, 80% O_2_+20% CO_2_) on mold-yeast count of minced water buffalo meat at 2°C±0.5°C for 14 days. CFU, colony-forming unit.

Packaging method and interaction of packaging method and storage time had a very significant effect (p<0.01) on mold and yeast. Storage time also had a significant effect (p<0.05) on mold and yeast counts of samples (Table 3). The HiOx-MAP samples exhibited a higher mold-yeast average count than VP samples. On the other hand, an increase of only 0.5 log units in mold-yeast count was observed at the end of storage (Table 3). As shown in Figure 6, vacuum packaging showed an inhibitory effect on the count during chilled storage. In contrast, a slight growth of mold-yeast was detected in HiOx-MAP samples. In a study conducted on minced beef by Lambropoulou et al [40], a similar result was found. Değirmencioğlu et al [35] found that there was no increase in the total count of mold-yeast in vacuum packaging. Also, Lorenzo and Gomez [27] reported that the growth of mold-yeast in vacuum samples was slower than MAP (80% O_2_+20% CO_2_) samples and the growth of mold-yeast decreased in both packaging methods after the 10th day.

The most important finding of the research is that lipid oxidation proceeds more rapidly than expected in minced water buffalo meat packed with HiOx-MAP method. Another finding is that a* value, which is one of the important criteria that influences consumer preference as a result of increase in TBARS value and which represents intensity of red color, started to decrease after the 6th day in HiOx-MAP application. In general, similar microbiological findings were obtained in both packaging methods. Moreover, the research also revealed that lipid oxidation is a good indicator for the shelf life of minced water buffalo meat packed with HiOx-MAP method. As a result, it can be said that lipid oxidation in minced water buffalo meat packed with HiOx-MAP can be delayed by using natural antioxidants and further research on this subject would be beneficial.

## Figures and Tables

**Table 1:** Effects of vacuum packaging (VP) and high-oxygen modified atmosphere packaging (HiOx-MAP, 80% O_2_+20% CO_2_) on pH and TBARS values of minced water buffalo meat at 2°C±0.5°C for 14 days (mean±standard deviation)

Factors	pH	TBARS
Packaging method (PM)		
VP	5.70±0.04^a^	0.16±0.04^a^
HiOx-MAP	5.71±0.04^a^	2.91±2.35^b^
Significance	NS	**
Storage time/d (ST)		
0	5.65±0.01^a^	0.13±0.02^d^
2	5.71±0.05^bc^	0.20±0.11^d^
4	5.70±0.02^b^	0.89±0.82^c^
6	5.69±0.01^b^	0.96±0.94^bc^
8	5.75±0.02^d^	1.38±1.46^b^
10	5.70±0.04^b^	2.80±2.88^a^
12	5.74±0.01^cd^	2.85±2.98^a^
14	5.70±0.03^b^	3.09±3.20^a^
Significance	**	**
PM×ST	NS	**

TBARS, thiobarbituric acid reactive substances; NS: not significant.

a–d Any two means in the same column having the same letters in the same section are not significantly different at p>0.05,

** p<0.01.

**Table 2:** Effects of vacuum packaging (VP) and high-oxygen modified atmosphere packaging (HiOx-MAP, 80% O_2_+20% CO_2_) on color (L*, a*, and b*) of minced water buffalo meat at 2°C±0.5°C for 14 days (mean±standard deviation)

Factors	L*	a*	b*
Packaging method (PM)			
VP	35.16±1.17^a^	21.88±1.54^b^	0.80±0.47^a^
HiOx-MAP	38.56±1.50^b^	18.67±7.34^a^	4.72±0.92^b^
Significance	**	**	**
Storage time/d (ST)			
1	37.50±2.00^bc^	24.40±3.54^d^	3.16±3.05^b^
2	36.41±1.90^ab^	24.10±3.38^d^	3.13±2.45^b^
4	36.34±2.90^ab^	22.85±2.42^d^	3.00±2.54^b^
6	35.60±1.64^a^	20.57±1.22^c^	2.50±1.54^ab^
8	36.53±2.23^ab^	20.66±2.70^c^	2.24±2.09^a^
10	37.36±1.71^bc^	18.32±5.62^b^	2.25±1.60^a^
12	38.32±2.67^c^	16.19±6.94^a^	3.00±2.19^b^
14	36.88±2.20abc	15.11±7.88^a^	2.80±2.15^ab^
Significance	*	**	*
PM×ST	NS	**	**

NS, not significant.

a–d Any two means in the same column having the same letters in the same section are not significantly different at p>0.05,

** p<0.01,

* p<0.05.

**Table 3:** Effects of vacuum packaging (VP) and high-oxygen modified atmosphere packaging (HiOx-MAP, 80% O_2_+20% CO_2_) on the microbiological counts (log CFU/g) of minced water buffalo meat at 2°C±0.5°C for 14 days (mean±standard deviation)

Factors	Total aerobic mesophilic bacteria	Psychrotrophic bacteria	*Pseudomonas*	Lactic acid bacteria	Enterobacteriaceae	Molds/yeasts
Packaging method (PM)						
VP	5.22±1.21^b^	5.56±1.35^a^	3.51±0.22^a^	5.00±1.38^b^	2.89±0.32^b^	3.16±0.21^a^
HiOx-MAP	4.97±0.98^a^	5.65±1.38^b^	3.66±0.30^b^	4.71±1.19^a^	2.66±0.38^a^	3.50±0.33^b^
Significance	**	*	*	**	**	**
Storage time/d (ST)						
0	3.79±0.09^a^	4.02±0.16^a^	3.51±0.20^ab^	3.44±0.34^a^	2.82±0.23^bc^	3.02±0.11^a^
2	4.07±0.09^b^	4.18±0.11^ab^	3.59±0.15^b^	3.41±0.21^a^	2.54±0.22^ab^	3.29±0.08^b^
4	4.17±0.09^b^	4.36±0.19^b^	3.60±0.28^b^	3.66±0.18^a^	2.31±0.35^a^	3.34±0.10^b^
6	4.59±0.31^c^	5.04±0.20^c^	3.63±0.32^b^	4.36±0.26^b^	2.64±0.29^b^	3.27±0.17^b^
8	5.29±0.23^d^	5.77±0.12^d^	3.77±0.16^b^	5.30±0.33^c^	2.71±0.32^b^	3.33±0.32^b^
10	5.64±0.11^e^	6.56±0.25^e^	3.28±0.28^a^	5.64±0.26^d^	3.02±0.24^cd^	3.40±0.36^b^
12	6.37±0.57^f^	7.27±0.14^f^	3.52±0.21^ab^	6.25±0.53^e^	3.01±0.30^cd^	3.44±0.36^b^
14	6.85±0.24^g^	7.61±0.12^g^	3.78±0.31^b^	6.80±0.14^f^	3.16±0.19^d^	3.54±0.61^b^
Significance	**	**	*	**	**	*
PM×ST	**	NS	NS	*	NS	**

CFU, colony-forming unit; NS, not significant.

a–g Any two means in the same column having the same letters in the same section are not significantly different at p>0.05.

** p<0.01,

* p<0.05.

## References

[1] Tateo A, De Palo P, Quaglia NC, Centoducati P (2007). Some qualitative and chromatic aspects of thawed Buffalo (*Bubalus Bubalis*). Meat Sci.

[2] Rey JF, Martínez CL, Urrea A (2011). Comparative study of the physicochemical characteristics of an economic Buffalo (*Bubalus Bubalis*) meat product and an economic Beef (*Bos İndicus*) meat product with incorporation of bovine hemoglobin in powder in both formulations. Procedia Food Sci.

[3] Nisar PUM, Chatli MK, Sharma DK, Sahoo J (2010). Effect of cooking methods and fat levels on the physico-chemical, processing, sensory and microbial quality of buffalo meat patties. Asian-Australas J Anim Sci.

[4] Devadason IP, Anjaneyulu ASR, Babji Y (2010). Effect of different binders on the physico-chemical, textural, histological and sensory qualities of retort pouched buffalo meat nuggets. J Food Sci.

[5] Zhang W, Naveena BM, Jo C, Sakata R (2017). Technological demands of meat processing-An Asian perspective. Meat Sci.

[6] Ahmad S, Srivastava PK (2007). Quality and shelf life evaluation of fermented sausages of buffalo meat with different levels of heart and fat. Meat Sci.

[7] Kaban G (2013). Sucuk and pastırma: Microbiological changes and formation of volatile compounds. Meat Sci.

[8] Sachindra NM, Sakhare PZ, Yashoda KP, Rao DN (2005). Microbial profile of buffalo sausage during processing and storage. Food Control.

[9] Ahmad S, Rizawi JA, Srivastava PK (2010). Effect of soy protein isolate incorporation on quality characteristics and shelf-life of buffalo meat emulsion sausage. J Food Sci Technol.

[10] Akköse A, Kaban G, Karaoğlu MM, Kaya M (2018). Characteristics of pastırma types produced from water buffalo meat. Kafkas Üniv Vet Fak Derg.

[11] Paleari MA, Beretta G, Colombo F (2000). Buffalo meat as a salted and cured product. Meat Sci.

[12] Limbo S, Torri L, Sinelli N, Franzetti L, Casiraghi E (2010). Evaluation and predictive modeling of shelf life of minced beef stored in high-oxygen modified atmosphere packaging at different temperatures. Meat Sci.

[13] Bao Y, Puolanne E, Ertbjerg P (2016). Effect of oxygen concenrration in modified atmosphere packaging on color and texture of beef patties cooked to different temperatures. Meat Sci.

[14] Yang X, Niu L, Zhu L (2016). Shelf-life extension of chill-stored beef longissimus steaks packaged under modified atmospheres with 50% O_2_ and 40% CO_2_. J Food Sci.

[15] Garcia-Lopez ML, Prieto M, Otero A, Davies A, Board R (1998). The physicological attributes of Gram-negative bacteria associated with spoilage of meat and meat products. The microbiology of meat and poultry.

[16] Moczkowska M, Poltorak A, Montowska M, Pospiech E, Wierzbicak A (2017). The effect of the packaging system and storage time on myofibrillar protein degradation and oxidation process in relation to beef tenderness. Meat Sci.

[17] Jeong JY, Claus JR (2011). Color stability of ground beef packaged in a low carbon monoxide atmosphere or vacuum. Meat Sci.

[18] Suman SP, Joseph P (2013). Myoglobin chemistry and meat color. Annu Rev Food Sci Technol.

[19] Sahoo J, Anjaneyuldu ASZ (1997). Quality improvement of ground buffalo meat by preblending with sodium ascorbate. Meat Sci.

[20] Arun KD, Anjaneyulu ASR, Biswas S (2006). Effect of carnosine preblending on the quality of ground buffalo meat. Food Chem.

[21] Naveena BM, Sen AR, Muthukumar M, Babji Y, Kondaiah N (2011). Effects of salt and ammonium hydroxide on the quality of ground buffalo meat. Meat Sci.

[22] Lemon DW (1975). An improved TBA test for rancidity new series circular. No: 51.

[23] SPSS (2011). IBM SPSS statistics base.

[24] Lauzurica S, Fuente JDL, Di Az MT (2005). Effect of dietary supplementation of vitamin E on characteristics of lamb meat packed under modified atmosphere. Meat Sci.

[25] Kim YH, Huff-Lonergan E, Sebranek JG, Lonergan SM (2010). High-oxygen modified atmosphere packaging system induces lipid and myoglobin oxidation and protein polymerization. Meat Sci.

[26] Jayasingh P, Cornforth DP, Brennand CP, Carpenter CE, Withtier DR (2002). Sensory evaluation of ground beef stored in high-oxygen modified atmosphere packaging. J Food Sci.

[27] Lorenzo JM, Gomez M (2012). Shelf life of fresh foal meat under MAP, overwrap and vacuum packaging conditions. Meat Sci.

[28] Murphy KM, O’Grady MN, Kerry JP (2013). Effect of varying the gas headspace to meat ratio on the quality and shelf-life of beef steaks packaged in high oxygen modified atmosphere packs. Meat Sci.

[29] Garcia de Fernando GD, Nychas GJE, Peck MW, Ordonez JA (1995). Growth/survival of psychrotrophic pathogens on meat packaged under modified atmospheres. Int J Food Microbiol.

[30] Grobbel JP, Dikeman ME, Hunt MC, Milliken GA (2008). Effect of packaging atmospheres on beef instrumental tenderness, fresh color stability and internal cooked color. J Anim Sci.

[31] Storia AL, Ferrocino I, Torrieri E (2012). A combination of modified atmosphere and antimicrobial packaging to extend the shelf life of beefsteaks stored at chill temperature. Int J Food Microbiol.

[32] Esmer OK, Irkin R, Degirmencioglu N, Degirmencioglu A (2011). The effect of modified atmosphere on microbiological criteria, color and oxidation values of minced beef meat. Meat Sci.

[33] Berruga MI, Vergara H, Gallego L (2005). Influence of packaging conditions on microbial and lipid oxidation in lamb meat. Small Rum Res.

[34] ICMSF (1986). International Commission on Microbiological Specifications for Foods. Microorganisms in foods. 2. Sampling for microbiological analysis: principles and specific application.

[35] Değirmencioğlu N, Esmer OK, İrkin R, Değirmencioğlu A (2012). Effect of vacuum and modified atmosphere packaging on shelf life extention of minced meat chemical and microbiological changes. J Anim Vet Adv.

[36] Gökalp HY, Kaya M, Zorba O (2012). Engineering of meat products processing.

[37] Lee KT, Yoon CS (2001). Quality change and shelf life of imported vacuum-packaged beef chuck during storage at 0°C. Meat Sci.

[38] Djordjevic J, Boskovic M, Dokmanovic M (2017). Vacuum and modified atmosphere packaging effect on *Enterobacteriaceae* behaviour in minced meat. J Food Process Preserv.

[39] Stella S, Bernardi C, Tirloni E (2018). Influence of skin packaging on raw beef quality: a review. Hindawi J Food Qual.

[40] Lambropoulou KA, Drosinos EH, Nychas GJE (1996). The effect of glucose supplementation on the spoilage microflora and chemical composition of minced beef stored aerobically or under a modified atmosphere at 4°C. Int J Food Microbiol.

